# Observations on a Case of Inflammation of the Veins, after Amputation, Resembling Phlegmasia Dolens

**Published:** 1826-07

**Authors:** G. J. Guthrie


### COMMUNICATIONS.

---

### *Observations on a Case of Inflammation of the Veins, after Amputation, resembling Phlegmasia Dolens.* By G. J. Guthrie, Esq.

Inflammation of the veins is the most fatal consequence of amputation, and most frequently occurs where the patient has previously been in a very irritable or suffering state. It is of two kinds,—the adhesive or healthy, and the irritative or unhealthy, embracing all the different shades of inflammation between them. The first kind is seldom observed when it takes place, and, when observed, is usually cured. The latter is almost as invariably fatal. I have seen it destroy—I had nearly said, hundreds; being, in fact, the most common cause of death after amputation. When a person, after undergoing this operation, is about to suffer from unhealthy inflammation of the veins, the pulse quickens, and continues above ninety, and generally from 100 to 130, until his dissolution; the stomach sickens; there is for the most part frequent attacks of vomiting, usually of a bilious character, followed by the common symptoms of fever; he is restless, sleepless, and anxious; the tongue white. There is, in general, after the first three or four days, some marked paroxysms of fever, with remissions (rigors). The patient emaciates rapidly; the skin becomes tinged of a yellowish hue, and often covered with perspiration; bowels irregular. The pulse becomes weaker and more irritable, and increases in frequency as the disease goes on. The patient gradually sinks; or the febrile symptoms subside, with the exception of the frequency of the pulse, which also may even be diminished; he rallies a little, and the appetite returns: but, whilst he says he is better, and will get well, the daily, nay almost hourly, deterioration of the appearance is well marked, and a slight accession of fever soon closes the scene.

The stump is not in more pain than in many other cases in which no inconvenience follows, and frequently there is neither more pain nor suffering than is common to the operation. Neither is there any remarkable pain or tenderness in the course of the vessels.

In the following case, the femoral vein, as high as Poupart's ligament, of the amputated or right side, was frequently examined by pressure, and the iliac above the ligament in the same manner; but it never gave more uneasiness than the same degree of pressure would have done during health; for the occurrence of inflammation was suspected from the sickness, and the state of the pulse, previously to the explosion of symptoms on the left side; no part of which, from the iliac region downwards, would admit of the slightest pressure without great suffering. The evolution of heat was considerable; the sudden and great enlargement of the whole of the superficial veins was early very conspicuous; which, with the severity of pain experienced at all times, and augmented to agony on the slightest motion, were the only perceptible differences, and those only in degree, to be observed between it and a case of phlegmasia dolens, either by comparison with what I recollected, or recollect of cases previously or subsequently seen. It is also the only instance, I have observed, out of more cases, than have perhaps fallen to the lot of most surgeons to see, in which the femoral veins of the opposite side became affected after amputation of the other extremity. I have seen the iliacs of the opposite side frequently implicated, but I do not remember a case of the inflammation having proceeded lower. In few can the inflammation be traced higher than the diaphragm, and the patient usually dies before it passes the point of junction of the emulgent veins.

This case may be viewed as one giving rise synthetically to phlegmasia dolens, from a similar, although not the same cause, occurring in a different manner. It tends to confirm analytically the etiology and pathology of this disease, as advanced by Dr. Davis, in his paper in the twelfth volume of the Medical and Chirurgical Transactions, viz. that phlegmasia dolens, occurring in puerperal women, is caused by an inflammation of one or more of the principal veins within, and in the immediate neighbourhood of, the pelvis." In them it is usually, although not always, a curable complaint; whilst inflammation of the veins after amputation is generally as fatal. The difference consists in this—that the inflammation in puerperal women is most frequently of the healthy or adhesive kind; whilst, after amputation, it is of the unhealthy or erysipelatous kind.

Dr. Davis, in stating that inflammation of the veins of and near the pelvis is the cause of phlegmasia dolens, conjectures that it takes place in them in consequence of their being previously disposed to it, from the inconvenience, pressure, and excitement, they sustained during the period of pregnancy; and he quotes from Mr. Wilson's Lectures on the Blood, an opinion in support of this theory, viz.—that "all the veins liable to much pressure, or to enlargement of diameter, during pregnancy, appear to be more or less predisposed to inflammation upon the sudden removal of those agencies, by the consummation of the act of parturition." This hypothesis obtains support from the fact stated in the different dissections related by Dr. Davis and others, that the uterus and its appendages were sound, or in their natural state. Without having the slightest intention to dispute the accuracy of the gentlemen who made these dissections, or to hazard even a doubt upon what is past, I would suggest for the future the propriety of tracing the veins from the common iliac of the affected side down to the uterus; and, when attention is particularly directed to this point, I have little doubt but the inflammation of the veins will be found to have begun at the uterus, and to have ascended along a continuous surface, until it implicated the veins of the extremity. The inflammation being in a great degree adhesive, and the uterus not quite in a healthy state, in the generality of cases in which an examination takes place after death, the traces of inflammation may be readily overlooked in this part and the veins leading from it, unless they are made objects of especial observation.

Jane Strangemore, aged twenty-eight, was admitted into the Westminster Hospital, September 24th, 1823, with an elastic swelling of the whole of the knee-joint, measuring twenty-seven and a half inches in circumference. The swelling began in the under part five months previously, and gradually attained its present size, attended during its formation by severe paroxysms of pain, which have for the last three weeks been so violent as to deprive the patient of rest. Her general health is good, and she has suckled a baby for the last seven months, a fine healthy child.

The thigh was amputated by Mr. Guthrie on Saturday, the 27th; the bone being sawn through just below the trochanter. She suffered a good deal of pain after the operation : an opiate was administered and repeated, and she passed a good night.

28th.—The pulse, which previously to the operation was eighty, had increased to 100: there is, however, little heat of skin, and she appears easy. Some aperient medicine and saline draughts to be given every four hours. Towards evening, the stomach became sick; she vomited a quantity of bilious matter: pulse 120. Three grains of calomel, and one of opium, followed by the common aperient mixture, were ordered, and an enema. The region of the stomach, to which part pain was referred, is to have applied to it equal parts of ether and laudanum.

29th.—Bowels well evacuated: pain and sickness subsiding.

30th.—Appears better. Stump dressed, and looking well. Pulse 120.

October 1st.—Better in all symptoms, but looking irritable and ill. No pain any where; no sickness; appetite good; pulse still quick.

4th.—The house-surgeon called up in the night, on account of pain in the stump, which was relieved by changing the dressings.

8th.—Two ligatures have come away. The wound looks well; the edges have nearly healed. Eats meat, and with a good appetite.

9th.—Not so well: pulse 120; skin hot; feels ill; complains of pain in the other leg and thigh, which disturbed her rest. Was well purged, and the leg fomented. Towards evening, the pain was principally felt in the calf and in the heel.

10th.—Pulse 130; tongue furred; vomiting again of bile. The pain in the thigh, extending upwards to the groin and downwards to the heel, is intolerable, particularly in the latter part. The thigh and leg much swelled, and tender to the touch, although without redness. The swelling elastic, yet yielding to the pressure of the finger, but not in any manner like an œdematous limb. Mr. Guthrie pronounced the disease this morning to be inflammation of the veins, extending from the opposite side; but, after a careful examination, and on pressure, no pain was felt in the course of the iliac vessels of that side; and the stump looked well, save at one small point, corresponding to the termination of the femoral vein.

17th.—The symptoms continued nearly the same during the week; the sickness of stomach, and purging of bilious matter, abating at intervals.

18th.—Is better, and the pain is diminished. She looks somewhat better, but is becoming thinner.

20th.—Less pain in the limb, which is swelled, tender to the touch; and all the superficial veins are very much enlarged. The groin more swelled and tender. Sickness gone, and her appetite returning, and is allowed good, nourishing, simple diet. The stump had been poulticed since the 9th, to promote suppuration.

25th.—During these five days, it was interesting to see the patient eat and desire solid food, and, in her extremely emaciated state, seem to enjoy it. The bowels occasionally deranged. Pulse always from 126 to 136. Is slightly jaundiced in colour, but declares she is better, and will get well.

Monday, 27th.—Gradually sunk in the evening, and died; the limb having every where diminished in size, except at the groin, where the swelling was more circumscribed, resembling the appearance of a chronic abscess approaching the surface.

On examination after death, the termination of the vein on the face of the stump was open, and in a sloughy state: above that, for the distance of four inches, and as high as Poupart's ligament, the inside of the vein bore marks of having been inflamed; but the inflammation seemed to have been of an adhesive character. Above that point the inflammation appeared to have been of an irritative or erysipelatous kind, had gone on to suppuration, and the vein was filled with purulent matter, lymph, and blood, partly coagulated, partly broken down. These appearances extended up the cava beyond the diaphragm, and traces of inflammation could be distinctly observed almost into the auricle. This disease had passed along the right internal iliac and its branches; it had descended along the left iliac vein, affecting its branches in the pelvis to the uterus, and along the limb to the sole of the foot. At the left groin, the iliac vein becoming femoral, was greatly distended with pus, apparently of a good quality; and, if the patient had lived a day or two longer, it would have been discharged by a natural effort, as in chronic abscess. The viscera were healthy.

During the last days of this woman's life, no blood was returned from the lower half of the body, unless by the superficial veins: yet she was comparatively easy, although of a yellow hue, emaciated to the utmost, so as to represent a living skeleton; and in this state, with a pulse at 130, craving for and eating a whole mutton-chop, and more, at a time, with the most death-like countenance it is possible to conceive.

